# The interrelationship between confidence and correctness in a multiple-choice assessment: pointing out misconceptions and assuring valuable questions

**DOI:** 10.1038/s41405-021-00067-4

**Published:** 2021-02-12

**Authors:** Renata Grazziotin-Soares, Coca Blue, Rachel Feraro, Kristen Tochor, Thiago Machado Ardenghi, Donald Curtis, Diego Machado Ardenghi

**Affiliations:** 1 USASK-University of Saskatchewan, College of Dentistry, Saskatoon, SK Canada; 2 Department of Stomatology, UFSM—Federal University of Santa Maria, Santa Maria, Brazil; 3 Department of Preventive and Restorative Dental Sciences, UCSF—School of Dentistry, San Francisco, CA USA

**Keywords:** Dental foundation training, Root canal treatment

## Abstract

**Introduction:**

The aim of this study was to better understand the interfaces of being correct or incorrect and confident or unconfident; aiming to point out misconceptions and assure valuable questions.

**Methods:**

This cross-sectional study was conducted using a convenience sample of second-year dental students (*n* = 29) attending a preclinical endodontics course. Students answered 20 multiple-choice questions (“basic” or “moderate” level) on endodontics, all of which were followed by one confidence question (scale). Our two research questions were: (1) How was the students’ performance, considering correctness, misconceptions, and level of confidence? (2) Were the questions valuable, appropriate and friendly, and which ones led to misconceptions? Four situations arouse from the interrelationship between question correctness and confidence level: (1st) correct and confident, (2nd) correct and unconfident, (3rd) incorrect and confident (misconception) and (4th) incorrect and unconfident. Statistical analysis (*α* = 5%) considered the interaction between (a) students’ performance with misconceptions and confidence; (b) question’s difficulty with correctness and confidence; and (c) misconceptions with clinical and negative questions.

**Results:**

Students had 92.5% of correctness and 84.6% of confidence level. Nine students were responsible for the 12 misconceptions. Students who had more misconceptions had lower correctness (*P* < 0.001). High achieving students had low confidence in their incorrect responses (*P* = 0.047). ‘Moderate’ questions had more incorrectness (*P* < 0.05) and less confidence (*P* = 0.02) than ‘basic’. All questions were considered valuable [for example, the ones that presented images or required a mental picture of a clinical scenario, since they induced less misconception (*P* = 0.007)]. There was no difference in misconceptions between negative questions and other questions (*P* = 0.96).

**Conclusion:**

Preclinical endodontic students were highly correct and very confident in their responses. Students who had more misconceptions had also the lowest performance in the assessment. Questions were valuable; but some will worth further improvement for the future. A multiple-choice assessment, when combined with confidence questions, provided helpful information regarding misconceptions and questions value.

## Introduction

The literature is inefficient to produce a unifying theory on confidence. Many concepts have been described, some of them, focusing on a more broad definition, and others, focusing on confidence in specific tasks. Shrauger and Schohn^1^ elaborated one of the most comprehensive conventions for understanding confidence. They highlighted sources of confidence—including judgments of actual performance—and stated that confidence is a trait detectable by others in social interactions and activities. Those authors observed how confidence (and confidence ratings) is a product of behavior that feeds into subsequent decisions to engage in a behavior again. As a result, they defined confidence as “perceived (assuredness) in competence, skill or ability”. Lately, authors^2^ have claimed that, the previous definition conceptually overlaps the notion of “self-efficacy” described by Bandura^3^ and Zimmerman^4^. Bandura’s self-efficacy comprises for sources (past achievement; comparison with others; what others tell you and feelings or physical states) and four mediating processes (cognitive; motivational; affective and selective).^3^ This overlap leads to, sometimes, the use of self-confidence as a framework for (or synonym of) self-efficacy.^5^

When we think in complete a specific task we may define confidence as the degree of certainty about handling something.^6^ It is important to remember that confidence is individualist and specific, influenced by different physical environments and conceptual contexts. Self-efficacy is more complex than confidence: when confidence is the degree of certainty in content/statements, self-efficacy is impacted by sensitive and emotional factors related to the certainty in those content/statements.^2^

Studies investigating the relationship on confidence and accuracy/correctness have found a positive relationship between being confident and being correct.^7^ In addition, the feeling of confidence, together with notions of self-concept, self-efficacy, and anxiety are reliable indicators of academic achievements.^8^

Inquiring about student’s confidence in assessments has been reported as a way to identify the different confidence-correctness interactions.^9–13^ Confidence and correctness in multiple-choice questions may interact in four ways, each with different implications and outcomes for student learning. First, when a student chooses the correct answer in an assessment and is confident that the information is correct, it reflects the calibration between confidence and correctness. This calibration is an important attribute for appropriate clinical decision-making and professional development.^7,14–16^ In a second situation, when the student chooses the correct answer but is unconfident about his/her decision, there is the possibility that the student is guessing the answer. If that was the case, educators may not be able to detect the student’s deficiency, in which could result in the faculty not giving appropriate feedback.^17^ The third situation would be when the student chooses an incorrect answer and is sure that the answer is the correct one. This characterizes a misconception. In this case, the student is less likely to seek additional opinions before a decision-making (in a health-related treatment planning, for instance) and, therefore, is unaware of his/her responsibility of adverse outcomes that can be induced in patients.^14^ Often, misconceptions are difficult to detect and are resistant to change.^18^ Students can ignore or distort new information that conflicts with their previous erroneous thinking.^19^ The fourth situation is when the student chooses the incorrect answer and, at the same time, is unconfident about the chosen answer. This situation shows that the student is uninformed about the concept, but, at least, is aware of his/her deficiencies. In this situation, the student is usually open-minded and receptive to learning.^9^

Understanding the interrelationship between confidence and correctness helps educators in identifying uninformed and misinformed students, and, the topics that induce the most misunderstandings. Consequently, educators can provide feedback to students regarding their specific lack of knowledge, for example, pointing out a misconception and an implied etiology.^14,17,20,21^ For students, this can encourage the development of self-reflection and foster critical thinking.^14,20^ For example, one studied used a confidence score algorithm in multiple-choice examinations whereby leaving a question unanswered (admitting being unsure) rewarded students fractional points.^12^ In addition to that, the combination of confidence questions with multiple-choice questions with the aim of validating and increasing the standards of questions in assessments is a potential advantage. It is known that writing high standard multiple-choice questions is efficient in enhancing the cognitive levels of assessments.^22–24^

Our previous investigations, conducted in the United States, have already explored dentistry students’ confidence in assessments and were able to identify misconceptions in different disciplines, and showed that students were overconfident.^14,20,21^ Following those investigations, we found important to understand how generalizable our earlier results are to other environments and contexts (Canada). Therefore, this study was conducted with a second-year class of dentistry students whose answered multiple-choice questions combined with confidence questions. This allowed us to better understand the interfaces of being correct or incorrect and confident or unconfident; aiming to point out misconceptions and assure valuable questions.

## Material and methods

The protocol for this study was reviewed and approved by the appropriated University Behavioral Research Ethics Board (Beh-REB App ID #50).

### Study design and sample

This cross-sectional study was conducted using a convenience sample of second-year dental students from a Canadian dental school attending a preclinical endodontics course. The entire class (*n* = 31 students: 16 women and 15 men) was invited to participate and those who agreed to join the study signed a consent form.

### Assessment instrument

The assessment instrument consisted of 20 multiple-choice questions on endodontics, all of which were followed by one confidence question. This research wanted to answer the following inquires: (1) How was the students’ performance, considering correctness, misconceptions, and level of confidence? (2) Were the questions valuable, appropriate and friendly, and which ones led to misconceptions?

Each multiple-choice question had four alternatives (a, b, c, and d) and only one option was correct. Questions were developed according to the subjects addressed in class and posted online. Preclinical endodontics embrace technical topics regarding root canal treatment: access opening, instrumentation, intracanal medication, irrigation and obturation. The 20 multiple-choice questions had been previously designated in “basic” or “moderate”, according to the judgment of two faculty members. Item analysis (such as, difficulty index, discrimination index or distracter efficiency) was outside the scope of this study.

The confidence question asked “how confident are you about the accuracy/correctness of your chosen answer?” The student should point one of the options displayed in a horizontal visual scale: very unsure, unsure, sure, or very sure. Further, these options were dichotomized as ‘confident’ (sure, very sure) and ‘unconfident’ (very unsure, unsure). Students were advised that confidence levels would be an important factor to consider; however, if they preferred not to answer the confidence question, their grades would not be affected. Faculty members were blinded to the students’ identification on the assessment until the end of the course.

Four situations arouse from the interrelationship between question correctness and confidence level: (1st) correct and confident, (2nd) correct and unconfident, (3rd) incorrect and confident (misconception), and (4th) incorrect and unconfident.

### Data analysis

Aiming to answer our first research inquiry, we measured the frequency/percentage of the four situations described above in relation to the student. Statistical analysis was performed for the following interactions: (i) students’ performance × misconceptions and (ii) students’ performance × confidence. Aiming to answer our second research inquiry, we measured the frequency/percentage of the four situations, in relation to the multiple-choice questions. Statistical analysis was performed for the following interactions: (i) questions level of difficulty × correctness, (ii) questions level of difficulty and correctness × confidence, (iii) negative questions × misconceptions, and (iv) mental scenario/clinical questions × misconceptions.

Data analysis was performed with Chi-square and Mann–Whitney’s tests using Open Source Epidemiologic Statistics for Public Health (OpenEpi) Version 3.01 (www.OpenEpi.com) and Jamovi (www.jamovi.org), respectively. Significance was set at *α* = 5%.

## Results

### Students’ performance (correctness, confidence, and misconceptions)

From the 31 students who were invited to the study, one did not agree to participate (did not sign the consent form and did not answer the confidence questions) and another was excluded from the analysis since he/she left many confidence questions unanswered; resulting in 29 participants. Nine students (31.03%) were responsible for the 12 misconceptions responses detected. Students who had more misconceptions had also the lowest performance/correctness in the assessment (Mann–Whitney’s test, *P* < 0.001). High achieving students (the ones with scores of 85–100% on the assessment) were unsure in their incorrect responses (this means that students with high performance had more ‘situation 4’ than the rest of them) (Chi-square test, *P* = 0.047). Table 1 shows some additional descriptive the findings in relation to the students.

**Table 1:** Correctness, confidence level, and other important findings in relation to the students.

Total		Findings in relation to the students (*n* = 29)
Correctness	92.5%	8 students answered 20/20 (100%) questions correct; 7 students 19/20 (95%); 8 students 18/20 (90%); and 4 students 17/20 (85%). 2 students had the lowest performance, getting only 16/20 (80%) questions correct. The 92.5% means that 537of all 580 answers were answered correctly.
Confidence Level	84.6%	Students pointed ‘very sure’ or ‘sure’ on confidence scale. The majority of the students were highly confident showing ≥70% of sureness on their answers. Only one student was confident in only 50% of his/her answers and two other students were 65% confident.
Other important findings	(i)	The eight students who answered 100% of the questions correctly were highly confident in their answers (85.62%), but they also presented the ‘situation 2’, i.e., some uncertainty (14.37%).
	(ii)	Nine students (31.03%) were responsible for the 12 misconceptions responses detected. Seven students had one misconception each (five in ‘moderate’ questions and 2 in ‘basic’ questions); one student had two misconceptions (in ‘moderate’ questions); and another had three misconceptions (also in ‘moderate’ questions). Students who had more misconceptions had also the lowest performance, getting only 16/20 (80%) questions correct (*P* < 0.001).
	(iii)	The majority of students were high achieving (more than 85% correctness in the assessment) and, if they had some incorrect response, they indicated unconfidence on that. For example, the eight students who answered 18/20 questions correct and graded 90% in the assessment, five scaled unsure/very unsure for their two incorrect answers (‘situation 4’) (*P* = 0.047).

### Value of questions

Tables 2 and 3 show the findings in relation to the multiple-choice questions. ‘Basic’ questions had 95.17% of all correct answers. ‘Moderate’ questions had 67.44% of all incorrect answers. Moderate questions (as opposed to basic questions) induced the majority of incorrect answers (Chi-square test: *P* < 0.05). In addition, moderate questions also prompted lower confidence level, even when students responded correctly (‘situation 2’) (Chi-square test, *P* = 0.02).

**Table 2:** Interrelationship between confidence and correctness and other important findings in relation to the questions.

Interrelationship between confidence and correctness	Total	Findings in relation to the multiple-choice questions
Correct answer + confident (Situation 1)	77.9%	This percentage represents 452/580 of the answers. Questions 1 and 7 (‘basic’ questions) had 100% of correct answers and 100% of confidence.
Correct answer + unconfident (Situation 2)	14.65%	This percentage represents 85/580 of the answers.
Incorrect answer + confident (Situation 3)	2.06%	This percentage represents 12/580 of the answers. Nine students were responsible for the 12 misconceptions. These misconceptions originated from five questions: four ‘moderate’ and one ‘basic’.
Incorrect answer + unconfident (Situation 4)	5.34%	This percentage represents 31/580 of the answers.

Each one of the 29 students answered 20 questions each (resulting in 580 answers).

**Table 3:** Topic addressed on the question, question designation (degree of difficulty attributed by faculty members), percentage (%) of correctness, confidence, and % for each ‘Situation’ (interrelationship between confidence and correctness).

Question number	Topic	Question designation (degree of difficulty attributed by faculty members)	% Correctness (correct answers)	% Confidence This percentage represents Situation 1 + Situation 3 (students were confident regardless if they answered the question correctly or incorrectly)	Correct answer + confident (Situation 1)	Correct answer + unconfident (Situation 2)	Incorrect answer + confident (Situation 3) Misconception	Incorrect + unconfident (Situation 4)
1	Endodontic instruments	Basic	100%	100%	100%	0%	0%	0%
2	Endodontic instruments	Moderate	100%	89.66%	89.66%	10.34%	0%	0%
3	Access cavity preparation	Basic	100%	96.55%	96.55%	3.45%	0%	0%
4	Access cavity preparation	Moderate	96.55%	89.66%	89.66%	6.90%	0%	3.45%
5	Access cavity preparation	Basic	93.10%	89.66%	89.66%	3.45%	0%	6.90%
6	Cleaning and Shaping	Moderate	96.55%	89.66%	89.66%	6.90%	0%	3.45%
7	Cleaning and Shaping	Basic	100%	100%	100%	0%	0%	0%
8	Dental Dam Isolation	Basic	96.55%	89.66%	89.66%	6.90%	0%	3.45%
9	Endodontic instruments	Moderate	69%	48.27%	31%	27.5%	17%	24%
10	Obturation	Moderate	96.55%	100%	96.55%	0%	3.45%	0%
11	Cleaning and Shaping	Basic	93.10%	82.76%	82.76%	10.34%	0%	6.90%
12	Endodontic instruments	Basic	93.10%	58.62%	58.62%	34.48%	0%	6.90%
13	Pulp Physiology	Basic	96.55%	71.41%	71.41%	24.14%	0%	3.45%
14	Access cavity preparation	Basic	86.20%	79.31%	71.41%	13.79%	6.90%	6.90%
15	Cleaning and Shaping	Moderate	96.55%	82.76%	82.76%	13.79%	0%	3.45%
16	Cleaning and Shaping	Moderate	100%	89.66%	89.66%	10.34%	0%	0%
17	Endodontic instruments	Moderate	93.10%	71.41%	71.41%	20.70%	0%	6.90%
18	Cleaning and Shaping	Moderate	86.20%	51.72%	44.82%	41.38%	6.90%	6.90%
19	Endodontic instruments	Basic	93.10%	71.41%	71.41%	20.70%	0%	6.90%
20	Access cavity preparation	Moderate	75.86%	44.82%	37.93%	37.93%	6.90%	17.24%

Each question was answered by *n* = 29 students.

Table 4 shows examples of multiple-choice questions and important findings demonstrating their value (in other words, if they were appropriate, understandable, and friendly). We classified as valuable all 20 multiple-choice questions. However, the redesign/improvement of some questions for future assessments would be interesting, for instance: the five questions with negatively worded stems had 3 misconceptions from the 144 responses that were possible. Comparing to the rest of the questions (9 misconceptions from the 426 possible responses), the negative ones seemed to be misleading. However, there was not statistical difference in misconceptions between negative questions and the rest of them (Chi-square test, *P* = 0.96).

**Table 4:** Examples of multiple-choice questions, which were part of the endodontic assessment and important findings indicating if the question is valuable (appropriate, understandable, and friendly).

Question 1	‘Basic’	You have just completed the root canal treatment (cleaning and shaping using hand files) of two teeth in the Lab: a maxillary central incisor and a maxillary first premolar. Then, you took notes to be able to obturate the root canals in the next session: Tooth 11 —Working Length (WL): 22 mm, Initial Apical File (IAF): # 30, Master Apical File (MAF): # 50, Last file used in the Step-Back procedure: #80 Tooth: 24 (2 canals)—Buccal Canal WL: 19 mm, IAF: # 15, MAF: # 30, Last file used in the step-back procedure: # 55. Palatal Canal WL: 18 mm, IAF: # 20, MAF: # 35, Last file used in the step-back procedure: #60. Following your notes, answer: What are the handle colors for the files #30, #15, #55, #20, #35, #80, #60 respectively? (a) Green, blue, red, green, red, black, and blue (b) Blue, white, red, purple, pink, gray, and green (c) Green, white, red, yellow, green, purple, and red □ (d) Blue, white, red, yellow, green, black, and blue	These questions had 100% of correct answers and 100% of confidence (‘Situation 1’). A simple recall and understanding of ideas. They were valuable questions, and very basic.
Question 7	‘Basic’	The apical stop should be developed slightly short of the apical foramen; approximately 1.0 mm has been suggested. This leaves a tiny remaining portion of the canal not properly cleaned of bacteria and/ or debris. This section of the canal should finally be cleaned, not shaped, using fine instruments, no. #10 or no. #15, to the radiographic apex (or slightly beyond it). This procedure is know as: (a) working length (b) apical patency□ (c) crown-down phase (d) step-back phase	
Question 16	‘Moderate’	Put the sentences in order according to the protocol of instrumentation for ProTaper Gold that is attached to the Lab wall: 1. Use F1, F2, and F3 to enlarge the apical third (F2 or F3 will be your Master Apical File (MAF), depending on the canal anatomy and depending on the Initial Apical File (IAF) 2. Create radicular access (coronal pre flaring) with SX (golden/short file) until it gets resistance 3. Determine the Working Length: taking an X-Ray with the largest hand file (this file should be a #15, #20, or #25) 4. Use shaping files S1 and S2: S1 with brushing strokes, until it gets resistance. S1 is not required to reach the working length. S2 with brushing strokes, up to the working length. 5. Pre-determine the tooth length: measuring from the reference point (incisal or occlusal edge) to the radiographic apex 6. Use the patency file (#10 or #15) 1 mm less than your pre-determined X-ray measurement 7. Irrigate (before/during/after) and use the patency file to maintain the glide path and to check the foramen patency (approximately 1.5 mm beyond working length) (a) 5, 2, 6, 3, 4, 1, 7 □ (b) 1, 2, 3, 4, 5, 6, 7 (c) 2, 1, 3, 4, 6, 5, 7 (d) 5, 2, 6, 3, 1, 4, 7	This question also had 100% of correct answers; however it had only 89.66% of confidence (‘Situation 1’). Here, a complex sequence/alternatives that requires connection between theory and clinic procedures proved to be valuable, even if with some uncertainty by the students.
Question 9	‘Moderate’	Mark the correct alternative regarding rotary Nickel-Titanium (Ni-Ti) instruments: (a) clinical usage leads to great macroscopic changes in the alloy (b) have low flexibility (c) are able to accommodate great stress without increasing the strain, which means that in severe curved canals the strain against the canal wall is low □ (d) to prevent “cyclic fatigue” (fracture by stress) you should not force the instrument in apical direction and to prevent “taper lock” (torsional fracture) you should keep the instrument moving (in-and-out).	Question 9 had the highest level of incorrect answers (31%,) and it also had the highest percentage of misconceptions (17%). A connection between and theory clinical expertise was necessary (cognitive connection that sometimes is not possible at the preclinical stage of the dental program). Even so, we classified this question as “valuable”. Question 18 also had misconceptions—it required the indication of the wrong alternative. Redesigning and improvement of negative questions for future assessments would be interesting.
Question 18	‘Moderate’	Choose the alternative that does not refer to the sodium hypochlorite (NaOCL)(bleach), irrigant of choice in endodontics. (a) it presents toxicity. If it is inadvertently extruded through the tooth apex, or fell in the patient’s skin severe accidents may occur (b) it has the ability to dissolve vital and necrotic tissue, by breaking down proteins into amino acids (c) it has antimicrobial action, because of its high pH (d) it reduces the microhardness of dentin, which helps to negotiate and to maintain patency in narrow, tortuous, and calcified canals. However it might cause dentin erosion □	
Question 20	‘Moderate’	Underextending the access cavity preparation may lead to: (a) a missed canal (b) fracture of endodontic instruments (c) discoloration of the crown (d) all of the above □	Question with a high percentage of incorrect answers (24.14%) and, also, uncertainty. However, the positive point was that questions like this, requiring a mental picture of a clinical scenario, induced less number of misconceptions (*P* = 0.007).
Question 5	‘Basic’	Look at the palatal view of the maxillary lateral below and mark the design that represents an ideal traditional outline form for the endo access: □	Two similar questions with different results. Question 5 had less incorrect answers and less uncertainty. Questions using images and questions that required a mental picture of a clinical scenario—had less number of misconceptions (*P* = 0.007). Those are ‘valuable questions’.
Question 14	‘Basic’	Which of the following is the ideal endo-access (tooth face and outline) for mandibular incisors? (a) buccal and oval (b) lingual and oval (c) lingual and triangular □ (d) buccal and triangular	

Correct answer is checked by □.

At last, questions connecting basic science principles to clinical application (as question 20 for instance), induced some unconfidence. Interesting, questions using images and questions that required a mental picture of a clinical scenario had less number of misconceptions compared to theoretical/descriptive questions (Fisher’s exact test, *P* = 0.007).

Table 5 summarizes the statistical analysis and shows the main results.

**Table 5:** Summary of statistical results considering the interaction between different variables.

		Statistical analysis	
Interaction	Research findings	*P* value	Test (*α* = 5%)
Students’ performance × misconceptions	Students with more misconceptions had also the lowest performance in the assessment	*P* < 0.001	Mann–Whitney
Students’ performance × confidence	High achieving students were unsure in their incorrect responses	*P* = 0.047	Chi-square
Questions difficulty × correctness	Moderate questions induced the majority of incorrect answers	*P* < 0.05	Chi-square
Questions difficulty × confidence	Moderate questions prompted lower confidence level questions, even when students responded correctly	*P* = 0.02	Chi-square
Questions negative × misconceptions	Negative questions had similar misconceptions than other questions	*P* = 0.96	Chi-square
Questions clinical/mental scenario/images × misconceptions	Questions using images requiring a mental picture of a clinical scenario had less number of misconceptions compared to theoretical/descriptive questions	*P* = 0.007	Fisher’s exact

## Discussion

This study used a multiple-choice assessment to better understand the interrelationship between being confident (or not) in a chosen answer. Our study used multiple-choice questions combined with confidence scales aiming to point out misconceptions and to assure having valuable, understandable and friendly questions. With the use of confidence scales, we acquired unique information on students’ learning, mainly detecting the ones who were more likely to think critically and identifying the ones who needed additional support. Also, this assessment/study provided us knowledge about the value of multiple-choice questions, helping to improve future assessments.

Even without having a traditional multiple-choice assessment (with no confidence scale) to act as a group comparison in our study, it was undoubtedly, that adding confidence scales provided a more complete assessment. Literature has proved that confidence-based assessment techniques integrate the selection of multiple-choice answers with the student’s self-perceived level of certainty and offer a middle ground between the traditional multiple-choice answer and a lengthy essay response. Research has discovered that confidence-based assessments provide a more comprehensive measure of a person’s knowledge, increases the retainability of learned material and identifies topics in which people are misinformed.^25–27^ In addition, confidence scales also helped us to determine the value of questions, since we could verify if faculty’s understanding on the question was in line with students’ understanding (“basic” or “moderate” level of difficulty).

Having learned from previous studies, we expected that students would exhibit high confidence levels.^13,21,28^ In addition to foreseeing the students’ overconfidence, we also anticipated high percentage of correctness. Indeed, as expected, we found preclinical endodontic students highly correct (92.5%) and very confident in their responses (84.6%). Students who had more misconceptions had also the lowest performance in the assessment (statistically significant difference). One reason for the high accuracy was the relatively straightforward assessment that we have chosen. The nature of preclinical instruction in our academic setting is technical and focused on *modus operandi*, on simple recall of information and on developing hand skills; which requires, therefore, an assessment in consonance with. We are aware that straightforward and uncomplicated assessments may sometimes not be the ideal test to perform a discrimination power analysis (which was outside the scope of this study) and may not provide sufficient insights to measure students’ knowledge.^29^ Another reason for students being highly accurate in their answers is that this study was conducted with a small class, where students were able to receive individual and personalized attention; and the same faculty member delivered all the theoretical content. A single lecturer eliminates the challenges of calibration that might occur when several mentors are involved in the course. Since non-calibrated mentors may not apply the same standardization in protocols, techniques and philosophies,^30,31^ it could raise students’ misinterpretations and misconceptions. On the other hand, it is important to remember that, one disadvantage of having only one tutor, might be that it does not favor different types of learners, if the tutor uses only one modality of teaching.

The calibration between correctness and confidence is the ideal situation that fosters clever decision-making in a dental clinical setting and positively contributes to professional growth.^14–16,21^ The literature has shown the importance of increasing confidence through learning. Authors, by using a mixed-methods approach, proved how changes in confidence have positively influenced the clinical practice of two cohorts of general dental practitioners—demonstrating the following results: the ability to undertake more complex patient treatment with confidence; a realization that previously unattainable skills were attainable; a greater appreciation of one’s limitations; and a greater sense of job satisfaction, which was reflected by patient acceptance and contentment.^32^

Nonetheless, other evidences point in favor of a “medium” confidence level, saying that high confidence is not always appropriate. In isolation, overconfidence may be seen as undesirable professional characteristic as it is linked to a lack of accuracy and potential reckless behavior.^13^ In our study, we found high achieving students (the ones with scores equal or higher than 85% on the assessment) were still unsure in their incorrect responses (statistically significant). This is a situation where being unconfident is somehow worthwhile. It shows that although students did not know the answer, they were aware of their uncertainty. In other words, they were able to self-reflect on their uncertainty. Authors have stated that uncertainty can favor apprenticeship.^9^ If these dental students move from preclinical to the clinical setting with a ‘medium confidence’ in unfamiliar topics they will be open-minded and more prompt to seek tutoring, which may avoid clinical errors, unnecessary procedures, and harmfulness to the patient.^14,21^

Some correct answers were linked to under-confidence (unsure or very unsure). If this situation occurs in a traditional multiple-choice assessment (without confidence questions), it may lead to mentors to believe that students know the answer, when they were actually guessing.^20,33^ In our assessment, because of the confidence scale, we were able to provide feedback on both incorrect and correct student responses.

‘Moderate’ questions (as opposed to ‘basic’ questions) induced the majority of incorrect answers. These questions also prompted lower confidence levels, even when students responded correctly. Both results were statistically significant. Question 20, for instance, is a ‘moderate’ level and ‘requires a mental picture of a clinical scenario’ (see Table 4 for more information). Although it is composed of a very simple statement, students needed to mentally create the situation about access opening, connecting theoretical and clinical information. As the students-participants were in the preclinical level, they did not yet have clinical experience. Therefore, it is natural that they still have difficulties in transferring information from a preclinical to a clinical setting. To overcome this challenge of interfacing theory and technique, one idea is to use videos showing real time endodontic procedures,^34^ or to provide preclinical students with the opportunity of ‘shadowing’ more advanced-level peers in the clinical setting. On the other hand, the statistical analysis showed that questions requiring clinical scenario induced significant less number of misconceptions—which, therefore, made us to consider these types of questions valuable. Another type of valuable question, deserving to be used in further assessments, was the one that employed images/drawings. Question 5, in comparison to question 14, (see Table 4) facilitated the understanding of an endodontic opening access design, having significant less misconceptions. Therefore, we assumed that visual questions may benefit dentistry students, since they usually encounter a professional work environment that exploits images (photos, videos, radiographs, anatomic features, graphical abstracts, schemes, etc.).

Adversely, we also found questions that will need to be revised and improved for next assessments. And, although there was not statistical difference in misconceptions between negative word stem and the other questions, we did find a considerable number of misconceptions in negative questions. It is already known that negative questions might be potentially ineffective and misinterpreted.^35,36^ In here, we noticed that this type of question induced error, independent of confidence.

The strengths of this current study included the use of authentic data from an academic assessment, which helps assure students were appropriately engaged. Also, we were able to provide personal feedback for each learner—mainly the ones who got misconceptions. Limitations include having a convenience sample and having results from just one assessment. Our results might not be generalizable to other institutions; however, the findings about the questions may have external validity, since they showed inherent positive and negative characteristics of endodontic preclinical questions. Also, the inherent imprecision of the measurement of the confidence level—that is always a multifaceted and subjective measurement;^37^ and the multiple-choice assessment—that might foster study habits consistent with superficial or strategic learning (only to answer the questionnaire correctly). When this happen, the student would interested in recognizing isolated facts rather than relationships or higher levels of learning.^38^

As conclusion, we found that preclinical endodontic students were highly correct (92.5%) and very confident in their responses (84.6%). Students who had more misconceptions had also the lowest performance in the assessment. All questions were considered valuable; but some will need further improvement. This study showed that understanding the interfaces between confidence and correctness grant us with the possibility to point out students’ misconceptions (guaranteeing unique feedback for each student) and to assure we have valuable questions for current and future endodontic preclinical assessments.
